# 3D designed battery-free wireless origami pressure sensor

**DOI:** 10.1038/s41378-022-00465-0

**Published:** 2022-11-30

**Authors:** Taeil Kim, Amirhossein Hassanpoor Kalhori, Tae-Ho Kim, Chao Bao, Woo Soo Kim

**Affiliations:** Additive Manufacturing Laboratory, School of Mechatronic Systems Engineering, Simon Fraser University, Surrey, V3T 0A3 BC Canada

**Keywords:** Electrical and electronic engineering, Structural properties

## Abstract

A pressure monitoring structure is a very useful element for a wearable device for health monitoring and sports biomechanics. While pressure sensors have been studied extensively, battery-free functions working in wireless detection have not been studied much. Here, we report a 3D-structured origami-based architecture sensor for wireless pressure monitoring. We developed an architectured platform for wireless pressure sensing through inductor-capacitor (LC) sensors and a monopole antenna. A personalized smart insole with Miura-ori origami designs has been 3D printed together with conductive 3D printed sensors seamlessly. Wireless monitoring of resonant frequency and intensity changes of LC sensors have been demonstrated to monitor foot pressure for different postures. The sensitivity of the wireless pressure sensor is tunable from 15.7 to 2.1 MHz/kPa in the pressure ranges from 0 to 9 kPa and from 10 to 40 kPa, respectively. The proposed wireless pressure-sensing platform can be utilized for various applications such as orthotics, prosthetics, and sports gear.

## Graphical abstract

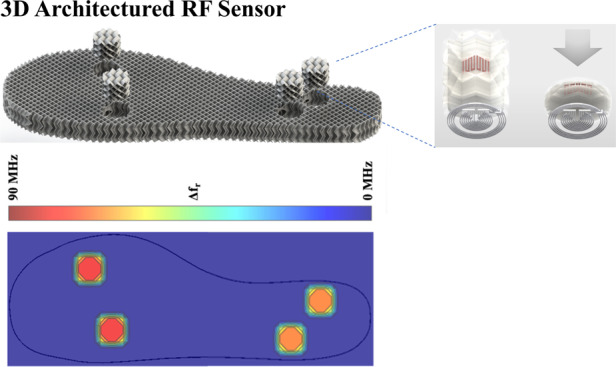

## Introduction

As precision medicine advances, there has been tremendous research works to develop wearable sensors to monitor physiological signals for the prevention, diagnosis, and treatment of diseases^1,2^. Biosensors can provide health information for early diagnosis of diseases such as respiratory diseases and heart diseases in the form of signals from specific parts of our bodies, such as pulse signals from the radial artery for arterial sclerosis, breathing signals from the respiratory muscles, and electrocardiogram from the chest^3,4^. Flexible pressure sensors have been developed for wearable applications based on sensing mechanisms such as capacitance, piezo-electricity, piezo-resistivity, and triboelectricity. Capacitive pressure sensors detect the pressure change based on the capacitance change caused by the change of distance of capacitor plates and have the advantages of fast response and high sensitivity^5–8^. Piezo-electric pressure sensors have a transduction mechanism based on piezo-electricity which means that positive and negative charges are separated in a piezo-electric material when an external force is applied and have the advantages of the easy acquisition of electrical signals and cost efficiency^9–12^. Piezo-resistive pressure sensors show the regular resistance change that comes from changing conductive paths of the composite depending on external forces and have advantages of simple structure and manufacturing process^13–16^. The triboelectric pressure sensors have been proposed to solve the power supply of the sensor. The performance of self-powered triboelectric pressure sensors has been improved by selecting advanced materials and designing efficient structures^17–20^. The measurement of plantar pressure can be used to understand the health condition of feet and make an early diagnosis of foot ulceration in type 2 diabetes patients and to advance footwear design and development, gait analysis, and sports biomechanics^21–24^. For example, plantar pressure measured from the heel, lateral mid-foot, and metatarsal can be used to understand the health of users’ feet as well as other parts of their bodies^25^. By measuring and analyzing plantar pressure, we can make the advancement of early diagnosis of diseases including diabetes, rheumatoid arthritis, and Parkinson’s disease by obtaining an auxiliary clinic indicator related to those diseases^26–28^. There are commercialized plantar detection platforms like the Zebris gait analysis system, which provides comprehensive plantar pressure distribution. However, these are not wearable technologies and have limitations for continuous monitoring^29,30^. For ambulatory monitoring, researchers developed wearable plantar pressure detection devices^31,32^. Various types of pressure sensors, such as capacitive, piezo-electric, piezo-resistive, and photo-electric pressure sensors were developed with different sensing mechanism^33^. The advancement of pressure sensors was realized by utilizing various materials such as piezo-electric materials, including poly (vinylidene fluoride) (PVDF), lead zirconate titanate (PZT), aluminum nitride (AlN), and ZnO, which demonstrate the high performance of pressure sensing^34–36^. Especially, β-phase PVDF has gained tremendous attention because of its reliable mechanical properties and excellent chemical stability^37^. Though researchers have developed wired foot pressure sensors, the wired connection of these sensors have made the structure of sensing devices complicated, while wireless connection makes the design simple and provides a comfortable experience for users. For example, the wired connection between the sensors and a signal acquisition circuit may cause the limitation of the user’s movement and sacrifice comfort, durability, and softness. On the contrary, wireless communication using Bluetooth or LC-based sensors provides more freedom to move users’ feet. Thus, wearable sensors based on wireless communication technologies have gained more attention in medical and biomedical applications as well as smart assistive robotics^38–45^. Wireless sensors can be categorized as active and passive devices based on the power supply methods. An inductor-capacitor (LC) sensor is one of the most widely used passive sensors^46^. LC sensors have the advantages of durability, compact design, and no requirement for power source^47,48^. Both the inductor and the capacitor of LC sensors can contribute to pressure detection. However, the quality factor of a capacitive-dominant wireless LC sensor decreases along with the pressure, which severely holds back the accuracy of measurement^49^. To address this issue, an inductor with sensitivity for pressure measurement can be utilized^50^. Origami, an ancient papercraft method uses predesigned patterns and makes deformable 3D structures from 2D sheets. As one of the most useful origami structures, Miura-ori has been widely studied and applied in various fields such as engineering and architecture. For example, frequency-selective surfaces utilize not only single-layer structures but also multi-layer mirror-stacked Miura-ori structures. The multi-layer Miura-ori structure prepared with flexible polymer materials shows significantly improved strength compared to single-layer paper-based origami structures^51^. Miura-ori structure has advantages such as high surface area, stretchability, and rigid foldability. By stacking Miura-ori structure in different ways, a spring-like truss structure is obtained with tunable mechanical properties. The deformation of Miura-ori structure under compression demonstrates predictable results instead of random deformation^52–54^. Origami structures have been widely used in sensing, packaging, protection, and impact energy absorption^55–58^. Especially Miura-ori structure has high strength and remarkable impact energy absorption capability (EAC)^59,60^. Researchers found that Miura-ori metamaterials showed better EAC compared to honeycomb from quasi-static compression tests^61^. A Miura-ori sheet absorbs much more energy during deformation compared to a monolithic plate^62^. Here we developed a novel 3D-printed flexible origami-inspired insole for far-field wireless plantar pressure sensing. A mirror-stacked Miura-ori multi-layer structure combined with LC sensors was 3D printed as a pressure-sensing device that detects mechanical deformation from electrical signals. This origami-based insole can undergo the weight of the user by customization, which can capture pressure ranging from low pressure to 50-80 kgs of human weight. Sensing components include the 3D printed LC sensor and the embedded inductor fabricated by multi-directional 3D printing for a far-field sensing range with higher sensitivity. One of the main benefits of using origami structures is that we can tune the mechanical properties of its structure by changing the design parameters of origami, such as thickness and unit cell size, easily and effectively. Four sensing positions were selected based on the average foot pressure distribution of healthy subjects. Two LC sensors are placed around the front foot, and the other two LC sensors are positioned around the heel, which can be used for the measurement of pressure distribution.

Recently, active wireless pressure sensing systems have been developed. However, their wireless communication unit and power sources make the device bulky and complex^63^. This can be improved by utilizing simple RF sensors. Insoles with RF sensors utilizing coupling between a ring antenna and LC sensors have been developed. In this case, it requires to move a ring antenna to individual positions of LC sensors to measure the pressure. It is not the most convenient approach and may generate errors in measurements. The benefits of a 3D-structured wireless pressure sensor embedded into the insole include personalized sensitivity control depending on different feet from person to person and convenience of measurement. Also, 3D printing technologies enable us to design an architectured insole with a customized range of pressure sensing for specific users. For example, the proposed design should consider factors such as weight, foot shape, and foot size of users. If the users have different weights, the origami design should be tuned accordingly to cover the required ranges of pressure sensing. When users have different sizes and shapes of feet, the architecture insole design should be adjusted accordingly. For this purpose, 3D printing is one of the best approaches to develop customized architectured insoles with pressure sensing.

## Results and discussion

### 3D architecture origami pressure sensor

**Fig. 1:**
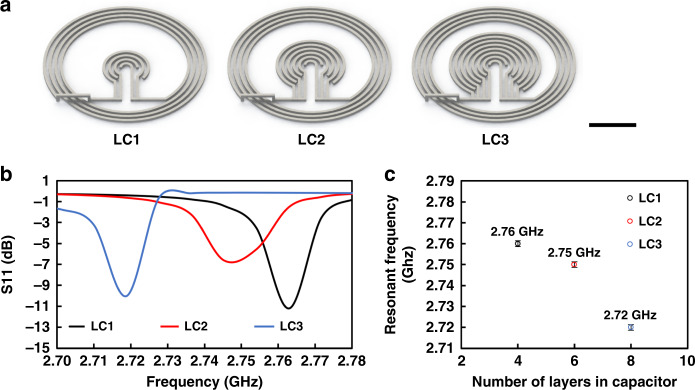
3D-designed LC sensors and their S11 simulation. **a** Three LC sensors have the same spiral inductor design with 3.75 turns, and outer diameter of 17.4 mm, and different interdigitated capacitors, which make different resonant frequencies (scale bar: 5 mm). **b** Simulated S11 shows peaks at resonant frequencies of LC sensors. **c** Resonant frequencies of LC sensors from the ANSYS HFSS simulations with different capacitors

Wireless origami pressure sensors are fabricated by multiple 3D printing technologies. The physical sensing of applied pressure at the origami structured sensing element generates the change of resonant frequency depending on the applied pressure. Three LC sensors with the same spiral inductors and different interdigital capacitors were designed as shown in Fig. 1a and fabricated by 3D printing of conductive silver ink on a polyimide (PI) substrate. The bridge that connects the most outer part of a spiral inductor and the central interdigital capacitor is designed in a horizontal direction. This bridge was printed on top of a dielectric layer that electrically insulates the bridge from the spiral inductor. Simulations of S11 were carried out to find resonant frequencies of three LC sensors.

The primary ring for the S11 reflection method sends an electromagnetic signal to the LC sensor. While the electromagnetic signal travels through the primary ring, the LC sensor resonates the most at its resonant frequency and shows a peak of the returned signal, as shown in Fig. 1b. The resonant frequency of LC sensors tends to decrease from 2.76 to 2.72 GHz as the number of layers in capacitor increases from 4 to 8 as shown in Fig. 1c. This agrees with the theory that describes the relationship between the resonant frequency and the capacitance of an LC circuit as given in Eq. 1.

1 $${{{\boldsymbol{f}}}} = \frac{1}{{2\pi \sqrt {{{{\mathbf{LC}}}}} }}$$

**Fig. 2:**
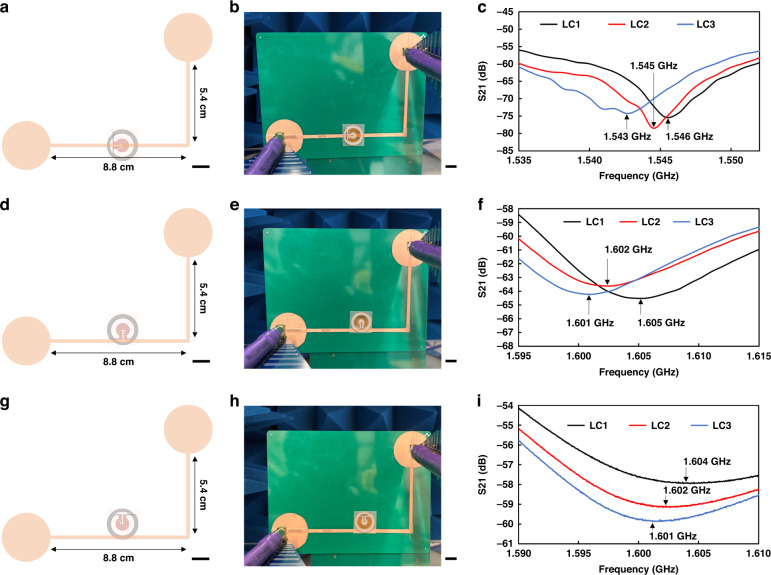
Experimental S21 test results with different orientations of LC sensor. The schematic (**a**) and the experimental setup (**b**) of LC2 with the bridge of LC to be perpendicular to the antenna. **c** S21 plot of the case of **a**. The schematic (**d**) and the experimental setup (**e**) of LC2 with the bridge of LC to be parallel to the antenna. **f** S21 plot of the case of **d**. The schematic (**g**) and the experimental setup (**h**) of LC2 with the bridge of LC to be parallel to the antenna. **i** S21 plot of the case of **g**. (all scale bars: 10 mm)

L-shaped antenna was used for wireless sensing with LC sensors. To understand the effect of the orientation of LC sensors with respect to the L-shaped antenna, three LC sensors were placed on an L-shaped antenna with different orientations to measure resonant frequency wirelessly using the S21 method. Figure 2a, d, g show the schematics of the L-shaped antenna with LC sensors with different orientations, and Fig. 2b, e, h show the experimental setup of S21 measurement of LC sensors with three different orientations with respect to the L-shaped antenna. Two commercial log periodic antennas with working frequency bands from 800 MHz to 6 GHz were connected to ports 1 and 2 of a vector network analyzer and fixed with an angle of 90 degrees to minimize the interference of signals. First, LC sensors were placed in the middle of the horizontal part of the L-shaped antenna with an orientation that the bridge of LC sensors is perpendicular to the horizontal part of the L-shaped antenna, as shown in Fig. 2a, b. Second, LC sensors were placed in the middle of the horizontal part of the L-shaped antenna with an orientation that the bridge of LC sensors is parallel and close to the horizontal part of the L-shaped antenna, as shown in Fig. 2d, e. Third, LC sensors were placed in the middle of the horizontal part of the L shape antenna with an orientation that the bridge of LC sensors is parallel to but apart from the horizontal part of the L-shaped antenna, as shown in Fig. 2g, h. LC sensors in three different orientations showed the same trend that the LC sensor with a higher capacitance showed a lower resonant frequency for all cases, as shown in Fig. 2c, f, i.

**Fig. 3:**
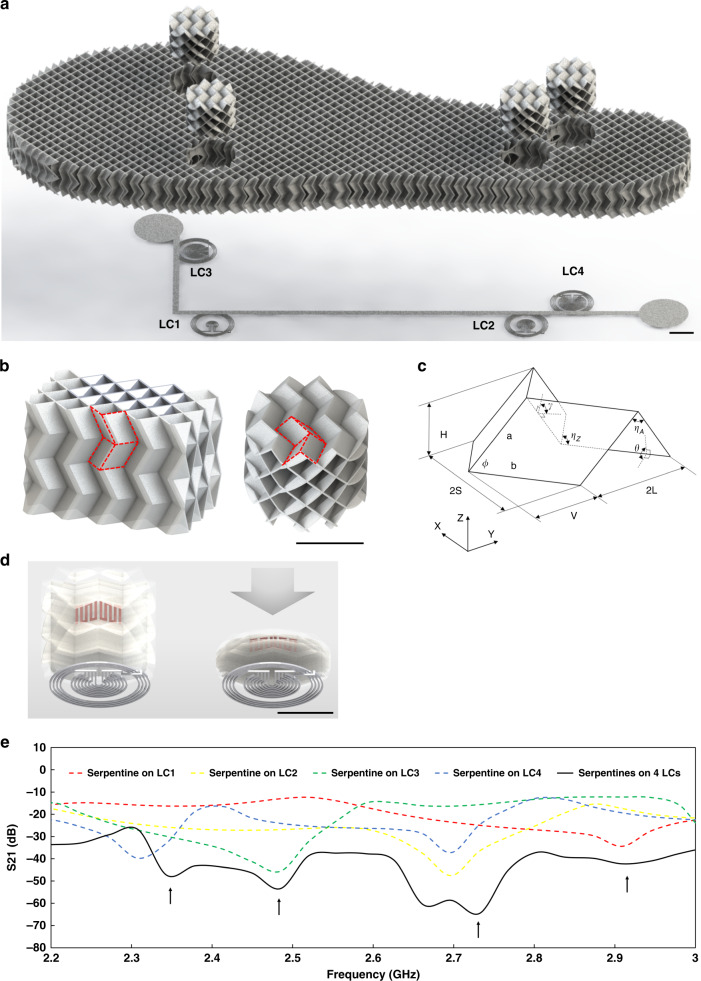
3D architectured insole design and simulation results of insole model. **a** An exploded view of the insole with four cylindrical origami blocks positioned on top of the four LC sensors (scale bar: 10 mm). **b** Descriptive schematics about the direction of origami for the overall insole part (left) and cylindrical pressure sensing part (right) (scale bar: 10 mm). **c** A typical Miura-ori foldcore. **d** 3D images of gap change when the origami block with conductive serpentines (pink) is compressed (scale bar: 10 mm). **e** S21 Simulation result of L-shaped antenna with only 1 pair of LC and serpentines (red, green, blue, and yellow), and L-shaped antenna with simultaneous measurement of four pairs of LCs and serpentines (black)

To make a wireless sensing insole that can detect the pressure applied, four LC sensors were embedded as pressure sensors in an insole with origami structures, as shown in Fig. 3a. LC1 is positioned under the head of the fifth metatarsal bone. LC2 is placed at the lateral border of the foot. LC3 is positioned under the head of the first metatarsal bone. LC4 is placed underneath the heel. By changing the orientation of the same origami structure, the insole was designed to show lower compressibility for applied pressure, and four origami blocks with cylinder shapes positioned on top of four LC sensors were designed to show higher compressibility. Four LC sensors are placed under the L-shaped antenna at the bottom of the insole.

The same origami structure was applied for the entire insole part and cylindrical shape pressure sensing components with different orientations to optimize the performance of pressure sensing of the insole by utilizing differing Young’s modulus of origami structures for different orientations. The insole design is based on multilayers of Miura-ori origami structure. The overall insole part has multiple layers of Miura-ori origami stacked in a perpendicular direction to the base plane of the insole (Fig. 3b left). It requires higher stress to achieve the same strain for the overall insole part compared to the cylindrical origami blocks, which have six layers of Miura-ori origami stacked in parallel to the base plane of the insole (Fig. 3b right). This cylindrical origami shows a lower Young’s modulus, which is 0.0134 MPa, as shown in Fig. S2. The origami of the overall insole shows a higher Young’s modulus, which is 0.443 MPa for the strain ranging from 0 to 0.1, and 0.634 MPa from 0.5 to 0.65. It shows a plastic buckling stage which mainly contributes to the significant energy absorption for the strain ranging from 0.1 to 0.5, as shown in Fig. S2b. This trend was also observed from the previous work^64^. By utilizing the combination of these two different orientations of origami effectively, it is possible to maximize the sensitivity of a plantar pressure sensor. The orientation which shows more flexible and easier compression contributes to the sensitivity of pressure sensors, while the other orientation with higher rigidity serves to resist the pressure from the weight of a human body.

Figure 3c shows the foldcore of the most widely studied Miura-ori origami pattern with four identical parallelograms, which is generated by folding a sheet. For the modeling of motions of this Miura-ori pattern, each face is considered as a rigid body, and the creases are considered as perfect line hinges. The side lengths are *a* and *b*, and the sector angle is *ϕ*. The dihedral angle *θ* defines the motion state of the unit. The below equations express the dimensions *V, L, S*, and *H* as well as the dihedral angle *γ* in terms of the dihedral angle *θ*.

2 $${{{\boldsymbol{S}}}} = {{{\boldsymbol{b}}}}\;{{{\boldsymbol{sin}}}}\phi \sin \left( {\frac{\gamma }{2}} \right) = \frac{{{{{\boldsymbol{s}}}}\sin \alpha \cos \theta }}{{\sqrt {1 - {{{\boldsymbol{sin}}}}^2\alpha {{{\boldsymbol{sin}}}}^2\theta } }}$$

3 $${{{\boldsymbol{L}}}} = \sqrt {{{{\boldsymbol{a}}}}^2 - {{{\boldsymbol{H}}}}^2} = {{{\boldsymbol{a}}}}\sqrt {1 - {{{\boldsymbol{sin}}}}^2\phi {{{\boldsymbol{sin}}}}^2\theta }$$

4 $${{{\boldsymbol{V}}}} = {{{\boldsymbol{b}}}}^2 - {{{\boldsymbol{S}}}}^2 = \frac{{2{{{\boldsymbol{ab}}}}\;{{{\boldsymbol{cos}}}}\phi }}{{{{\boldsymbol{L}}}}}$$

5 $${{{\boldsymbol{H}}}} = {{{\boldsymbol{a}}}}\sin \phi \sin \theta$$

To describe the mechanical characteristics related to the strength and energy absorption capacity of a Miura origami unit cell under the compression in X direction, Zhang et al. proposed an analytical model of the quasi-static response. The below equations are expressions of the quasi-static compression force *F*_*c*_ and specific energy absorption (SEA)^56^.

6 $${{{\boldsymbol{Fc}}}} = \sigma {{{\mathbf{m}}}}{{{\boldsymbol{t}}}}^2\frac{{\sqrt {(1 + {{{\boldsymbol{cos}}}}^2\theta {{{\boldsymbol{tan}}}}^2\phi )} }}{{{{{\mathbf{tan}}}}\phi }}\left[ {\frac{{\sqrt {\left( {1 + {{{\boldsymbol{cos}}}}^2\theta {{{\boldsymbol{tan}}}}^2\phi } \right)} }}{{{{{\mathbf{sin}}}}\theta }} + \frac{{{{\boldsymbol{a}}}}}{{{{\boldsymbol{b}}}}}\frac{1}{{\sqrt {{{{\boldsymbol{cos}}}}^2\phi \left( {1 + {{{\boldsymbol{cos}}}}^2\theta {{{\boldsymbol{tan}}}}^2\phi } \right) - {{{\boldsymbol{cos}}}}^2\theta } }}} \right]$$

7 $${{{\boldsymbol{W}}}} = 2{{{\boldsymbol{b}}}}\;{{{\boldsymbol{tan}}}}\left[ {\frac{{{{{\boldsymbol{cos}}}}\;\theta }}{{\sqrt {\left( {1 + {{{\boldsymbol{cos}}}}^2\theta {{{\boldsymbol{tan}}}}^2\phi } \right)} }} - \frac{{{{{\boldsymbol{cos}}}}\;\theta 0}}{{\sqrt {\left( {1 + {{{\boldsymbol{cos}}}}^2\theta 0{{{\boldsymbol{tan}}}}^2\phi } \right)} }}} \right]$$

8 $${{{\boldsymbol{SEA}}}} = \frac{{\sigma {{{\mathbf{m}}}}}}{{\rho {{{\mathbf{m}}}}}}\frac{1}{{\rho 0\cdot {{{\mathbf{A}}}}\left( {\theta 0} \right)\cdot 2{{{\boldsymbol{S}}}}(\theta 0)}}\mathop {\int }\limits_0^{|{{{\boldsymbol{Wf}}}}|} \frac{{{{{\boldsymbol{Fc}}}}\left( \xi \right)}}{{\sigma {{{\mathbf{m}}}}}}{{{\boldsymbol{d}}}}\xi$$

As the 3D printing parameters such as layer height affect the mechanical properties of 3D printed parts, two types of origami blocks with the printing layer height of 100 and 200 μm were prepared by 3D printing the same origami structure (*a* = 3.65 mm, *b* = 4.68 mm, *V* = 3.09, *L* = 7.36, *S* = 4.13 mm) considering printability and pressure applied. When these origami blocks with a thickness of 16 mm are compressed by 10 mm, the average maximum force was measured to be 171.8 N (printing layer height of 200 μm) and 37.3 N (printing layer height of 100 μm), respectively. The calculated pressure based on the area of blocks was 282.7 and 52.3 kPa, respectively. And if we calculate the pressure on the insole based on the weight of a person (80 kg) and the average contact area of the foot (80 cm^2^), it is 49.04 kPa if the person stands on two feet and 98.07 kPa if the person stands on one foot. Table 1 shows pressure applied under the foot for two cases of standing with 1 foot or 2 feet with differing weights. The origami blocks 3D printed with a layer height of 200 μm can cover all weights from 50 to 80 kg in case of standing on 2 feet. The origami blocks 3D printed with a layer height of 100 μm can cover all weights from 50 to 80 kg in case of standing on 1 foot. Force and stroke curves of origami blocks with a printing layer height of 100 and 200 μm show the typical load-displacement relationship of the energy-absorbing structures as shown in Fig. S3^59^.

**Table 1:** Pressure applied on the area of 1 foot or 2 feet depending on the user’s weight

Weight (kg)	Pressure - Standing on 2 feet (kPa)	Pressure - Standing on 1 foot (kPa)
50	30.65	61.29
60	36.78	73.55
70	42.91	85.81
80	49.04	98.07

According to CDC, the 3rd, 50th, and 97th percentile weight of 20 years old females is 45.05, 58.22, and 89.04 kg, respectively, and 3rd, 50th, and 97th percentile weight of 20 years old males is 54.00, 70.60, and 100.78 kg, respectively^65^. The pressure applied on the insole based on the 50^th^ percentile weight of 20-year-old females (58.22 kg) and males (70.60 kg) is 35.66 and 43.24 kPa, respectively, in case of standing on two feet, and 71.32 and 86.48 kPa, respectively in case of standing on one foot when the average contact area of the foot is 80 cm^2^.

The cylindrical block of the origami structure has a conductive serpentine part on the surface, as shown in Fig. 3d, which is fabricated with multi-directional 3D printing, as shown in Supplementary Video 1. As the origami block is compressed, the conductive serpentine gets closer to the LC sensor. This gap change between the LC sensors and serpentines generates the change in capacitance and inductance of the LC sensors and serpentines, which will change the resonant frequency of the circuit. Figure 3e shows the results from the simulations. For the same LC sensors and the L-shaped antenna in the insole, serpentines were placed on LC sensors individually and all together. When an individual serpentine was placed either on LC1, LC2, LC3, and LC4, four distinctive peaks were observed. When four serpentines were placed on LC1, LC2, LC3, and LC4, respectively, a plot with four peaks was obtained (black).

**Fig. 4:**
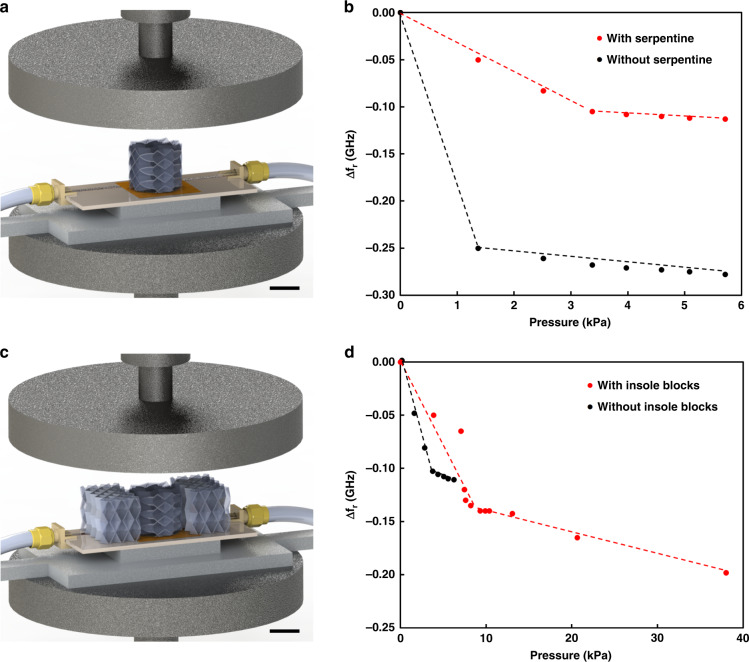
Sensitivity testing of origami pressure sensors with or without surrounding origami. **a** The experimental setup of compression of origami block placed on top of the LC4 (C9) (scale bar: 10 mm). **b** Resonant frequency changes (Δ f_r_) depending on applied pressure for LC4 with no insole blocks (red: with serpentine, black: without serpentine). **c** The experimental setup of compression of origami block together with two different insole parts placed on top of the LC4 (scale bar: 10 mm). **d** Resonant frequency changes (Δ f_r_) depending on applied pressure with serpentine (red: with insole blocks, black: without insole blocks)

To understand how the applied pressure changes the resonant frequency of LC sensors, a compression test for the cylindrical origami block was investigated, as shown in Fig. 4. The cylindrical origami block was placed on top of the LC sensor and a strip antenna to observe the change of resonant frequency of the LC sensors using S21 method as shown in Fig. 4a. The 3D printed Miura-ori structure shows repeatable and reliable strain-stress behaviors. As the origami block is highly compressive, it can be almost fully compressed with a pressure of less than 6 kPa. Two other cases of cylindrical origami blocks with and without serpentine were tested by the same compression. The common part of the results is that both cases show higher sensitivity for the lower pressure range and lower sensitivity for the higher pressure range. The serpentine contributes to a larger range of pressure that shows higher sensitivity, as shown in Fig. 4b.

As the origami structures of the sensing region and other parts of the insole were designed to have different orientations of origami structure, the cylindrical block together with two rectangular origami blocks with the same orientation as the insole were compressed by the compression tester, as shown in Fig. 4c. By adding two origami blocks which have the same structure as the insole on two sides of the cylindrical origami block, the pressure monitoring range was increased. The upper limit of the pressure monitoring range is 38.0 kPa, which is the maximum pressure applied to the Miura-ori structure when it is fully compressed. The LC sensor shows higher sensitivity (−15.7 MHz/kPa) in the pressure monitoring range up to 9 kPa. For the pressure range from 10 to 40 kPa, the LC sensor shows a lower sensitivity (−2.1 MHz/kPa), as shown in Fig. 4d.

**Fig. 5:**
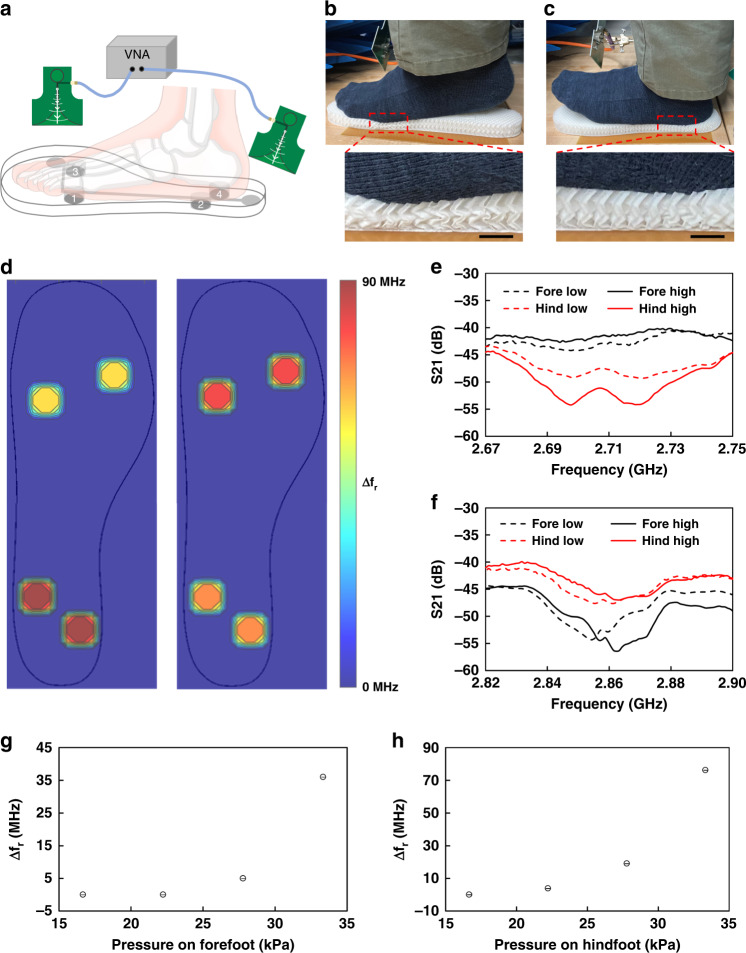
Wireless pressure sensing with a 3D architectured insole. **a** A schematic for the experiment setup of the insole with four different locations of sensor blocks and two antennas (green) connected to two ports of the VNA. **b**, **c** The photos of an experimental setup for the measurement of S21 of two cases. **b** Deformed insole with pressure on the forefoot. **c** Deformed insole with pressure on the hindfoot (scale bar: 10 mm). **d** Pressure mapping of two types of postures with a high pressure applied to the hindfoot and a low pressure to the forefoot (left) or a high pressure to the forefoot and a low pressure to the hindfoot (right). **e** Superimposed plot of S21 vs. Frequency for four cases; (1, 2) giving low and high pressure in the forefoot position of the insole, (3, 4) giving low and high pressure at the hindfoot position of the insole. **f** Superimposed plot of S21 vs. Frequency for four cases; (1, 2) giving low and high pressure in forefoot position of the insole, (3, 4) giving low and high pressure at the hindfoot position of the insole. **g** Resonant frequency changes (Δf_r_) depending on the pressure applied on forefoot position. **h** Resonant frequency changes (Δf_r_) depending on the pressure applied on hindfoot position

Finally, the 3D architectured insole integrated with four LC sensors and four cylindrical origami blocks was tested by stepping on it with different postures. Figure 5a shows the experimental setup to measure S21 as the different levels of pressure are applied in the forefoot or hindfoot area. The distance between the insole and a UWB antenna was set as 6 cm to obtain reliable resonance curves considering the limit of readout distance of 10 cm that was confirmed experimentally. The readout distance can be extended by increasing the size of the circular monopole at the bottom of the insole. However, the circular monopole was designed here to have a diameter of 2 cm to be fit into the insole. The centerline of a UWB antenna is directed at the center of the circular monopole at the bottom of the insole. The angle between the centerline of a UWB antenna and the bottom plane of the insole is set to be 45 degrees to have enough room for the leg and foot. The orientation of UWB antennas at the side and hind was set to be parallel to the corresponding straight lines of the L shape antenna, as shown in Fig. 5c.

Figure 5b, c show the cases that the pressure is focused on the forefoot area or hindfoot area of the insole, respectively. To understand the trend of S21 data depending on the pressure applied, low and high pressure were applied for both forefoot and hindfoot positions. Figure 5d shows the pressure contour of two different postures by visualizing the resonant frequency change from the initial state. The left image visualizes a high pressure applied to the hindfoot and a low pressure to the forefoot. Then, the right image represents a high pressure applied to the forefoot and a low pressure to the hindfoot. As shown in Fig. 5e, f, applying low and high pressure to forefoot and hindfoot positions show changes in the Q factor and their resonant frequency. The Q factor from Fig. 5f is calculated as 168.4. This Q factor can be further improved by using conductive ink materials with higher conductivity. The signal-to-noise ratio calculated from the ratio of mean to the standard deviation in the range of frequency from 2 to 3 GHz is 113 on average.

The architecture insole was also validated with known weights of 15, 20, 25, and 30 lb. placed on forefoot and hindfoot positions on top of a rectangular plastic block with 5 cm width, 8 cm length, and 1 cm thickness. As expected, the resonant frequency of LC sensors was increased as the pressure is increased by increasing the weight, as shown in Fig. 5g, h. The resonant frequency change is higher in the case of applying pressure on the hindfoot position as the insole structure around the hindfoot is narrower compared to the insole structure around the forefoot position, which contributes to a larger level of compression with the same pressure applied. The relationship between pressure and frequency change of pressure sensing blocks showed highly repetitive results with a standard deviation under 0.002 for the resonant frequency changes, as shown in Fig. 5g, h.

The detailed fabrication is described in the Materials and methods section.

## Conclusion

Here, we have developed a novel wireless origami-based pressure sensing platform using multiple 3D printing technologies, including FFF for the flexible insole, DIW for LC sensors, and multi-directional 3D printing for serpentines. Prior studies have demonstrated various plantar pressure sensing systems for footwear development, gait analysis, early diagnosis, and sports biomechanics^21–25^. However, a simple far-field RF pressure sensing platform has not been studied widely. Inspired by 3D printed generic architectured solids^66^, we chose Miura-ori origami design which is also a mechanically transferrable architectured solid to realize a reliable wearable pressure sensing element for continuous pressure monitoring.

After confirming the multiple identification of designed RF sensors, we have built a wireless pressure sensing system by 3D printing an insole with a monopole antenna and LC sensors at the same time. Specifically, we applied 3D-printed Miura-ori origami designs for wireless plantar pressure. This origami is designed for energy absorption which gives benefits to users for shock absorption. The sensitivity of the wireless pressure sensor is −15.7 MHz/kPa and −2.1 MHz/kPa in different ranges of pressure from 0 to 9 kPa and from 10 to 40 kPa, respectively, which can be tailored by changing the design parameters of origami structures. Hence, it is possible to monitor plantar pressure with the personalized insole that contains origami designs for shock absorption. This origami structure works as the main part of a pressure sensor as well as the entire structure of the insole that sustains the user’s weight. The proposed 3D origami-based pressure sensing element can be used for a wide pressure sensing range and personalized use. By utilizing several 3D printing technologies, we realized a cost-efficient customized insole which can contribute to personalized health monitoring. We envision that our work enables health monitoring and early diagnosis of diseases conveniently through wireless pressure sensing. The 3D-printed origami pressure sensor can be used to monitor foot health as well as sports biomechanics. The demonstrated 3D printed origami pressure sensor system utilizes RF communication as a signal transmission of pressure sensing, which doesn’t require any battery-powered devices.

## Materials and methods

### Fabrication of LC sensors

LC sensors were fabricated by using Musashi Direct Ink Writing (DIW) 3D printing system. A spiral inductor and interdigital capacitor were 3D printed and connected to make a closed circuit through a bridge over the inductor. The outer end of the spiral inductor was connected to the interdigital capacitor. LC sensors were printed with a silver nanoparticle-based conductive ink, Flex 2 (Voltera), using a 3D printing system (SHOT mini 100Sx and ML-808GX, Musashi Engineering, Inc.). The LC circuit was printed by the DIW extrusion printing of conductive ink on top of a 0.1-mm-thick PI substrate. After printing the inductor and capacitor of an LC sensor, it was dried overnight and cured at 100 °C for 30 min on a hotplate. The thickness and width of the LC sensor trace are 300 μm each. The conductivity of LC sensor traces is about 7.9 × 10^4^ S/cm from the information of resistivity data (Voltera, Flex 2 ink) with a resistivity of 1.265 × 10^−7^ Ωm. A dielectric layer was printed on top of the spiral inductor at the position of the bridge and cured, and the bridge was printed using the same conductive ink on top of the dielectric layer to connect the inductor and capacitor, making a closed circuit. This bridge was designed to be durable with a thickness of 300 μm and was 3D printed with mechanically flexible ink (Voltera, Flex 2 ink) with improved bending performance to minimize the damage from the repetitive application of pressure.

### Fabrication of insole

FFF-based 3D printing method is used to make the insole. Tenlog printer (3D Solutions) equipped with two nozzle extruder systems is used to fabricate the lattice structures of the flexible insole using Ninjaflex TPU filament (NINJATEK), which has a diameter of 1.75 mm. The printed insole has dimensions of a length of 27.3 cm and a width of 9.7 cm. The thickness of the insole is 17 mm, which is consisted of 85 layers of 3d printed layer with a height of 0.2 mm. The first layer of the insole was printed with a lower speed of 1.5 mm/sec to prevent a problem of the print not sticking to the bed. The rest of the insole was printed at a higher speed of 6 mm/sec. For the print setup, the layer height was set as 0.2 mm, and the infill density was set as 100%. For the nozzle setting, the nozzle size was set as 0.2 mm. The nozzle temperature was set as 220 °C. A 0.2 mm brass nozzle for thermoplastic filament is used with 200 μm printed layer resolution. After the insole is completed, an L shape antenna with a width of 2.5 mm for the straight parts and two circular monopoles with a diameter of 2 cm was printed at the bottom of the insole using Musashi Direct Ink Writing (DIW) 3D printing system.

### Experimental setup for wireless detection of the resonant frequency of LC sensors

For the wireless detection of the resonant frequency of LC sensors, a couple of log periodic antenna were connected to a Vector Network Analyzer (R&S ZND) to measure the reflection coefficient S21 and find the resonant frequency as shown in Fig. 2b, e, h. The distance between the log periodic antenna and the monopole was 2 cm.

### Experimental setup for wireless pressure sensing

For the wireless pressure sensing with insole and LC sensors, a couple of ultra-wide-band high-gain directional antenna was connected to two ports of Vector Network Analyzer (R&S ZND) to measure the reflection coefficient S21 and find the magnitude change and resonant frequency shift. The working frequency range of the antenna is 1.4–10.5 GHz. This high-gain antenna is a linearly polarized antenna. The voltage standing wave is less than 2.0, and the return loss is 10 dB. Each antenna was aligned in the direction of a straight line of L shape antenna, and the distance between the end of the antenna and the monopole of the insole was 5 cm.

### Research ethical approval

All procedures to perform the studies of the plantar pressure monitoring have been approved by the research ethics committee at Simon Fraser University in Canada: Minimal Risk Approval 2020s0033. A male volunteer with no prior medical history of physical disability was recruited for participation in this test. The signed consents were obtained from the individual. According to the legal term of Canada, the confidentiality of participants is strictly respected.

## Supplementary information

supplementary document

supplementary video 01
