# Supplementary material for: 3D designed battery-free wireless origami pressure sensor
Source: Microsyst Nanoeng. 2022 Nov 30;8:120. doi: 10.1038/s41378-022-00465-0 (PMC9708697; doi:10.1038/s41378-022-00465-0)
Supplement: Supplementary file 1 — supplementary document [file 41378_2022_465_MOESM1_ESM.docx]

**Supplementary Video 01.**

Serpentine conductive traces inside of valley in the origami structure fabricated by the multi-directional conductive printing technology.


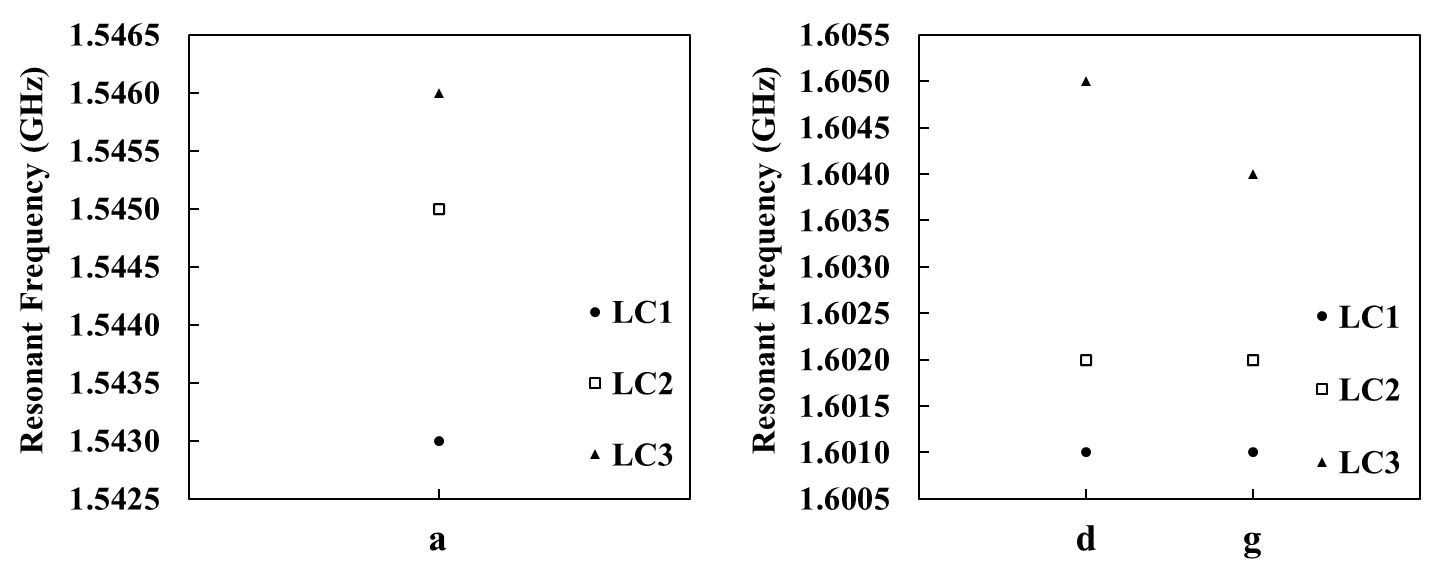


**Figure S1**. **Resonant frequencies of LC sensors from Figure 2c, f, and i**

**
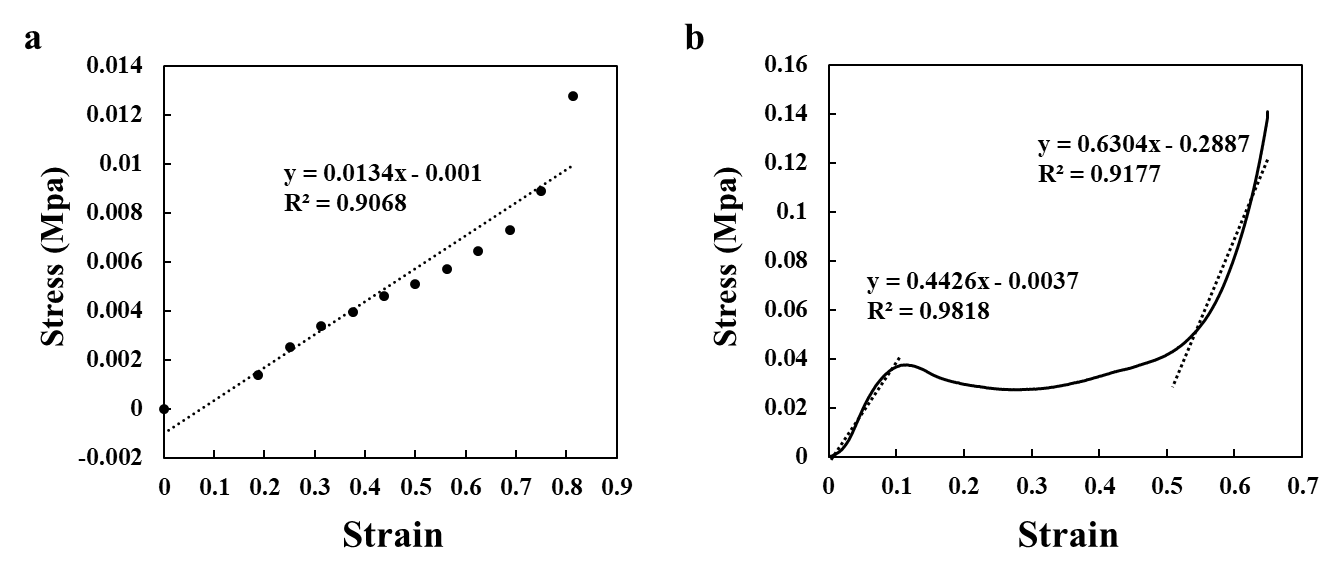
**

**Figure S2 Stress strain curves for Miura-ori origami blocks with different orientations** a) multiple layers of Miura-ori origami stacked in a perpendicular direction to the base plane of the insole b) multiple layers of Miura-ori origami stacked in parallel to the base plane of the insole.

**
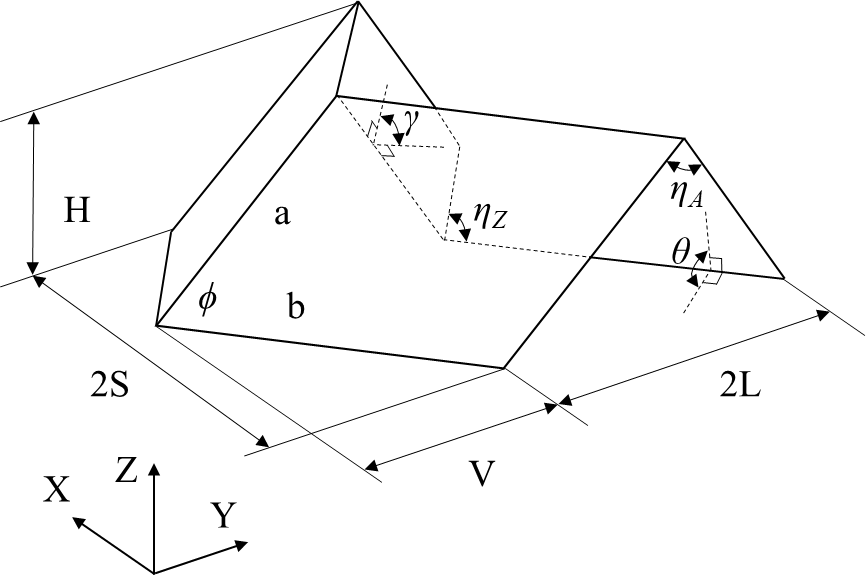
**

**Figure S3. A typical Miura-ori foldcore**

**
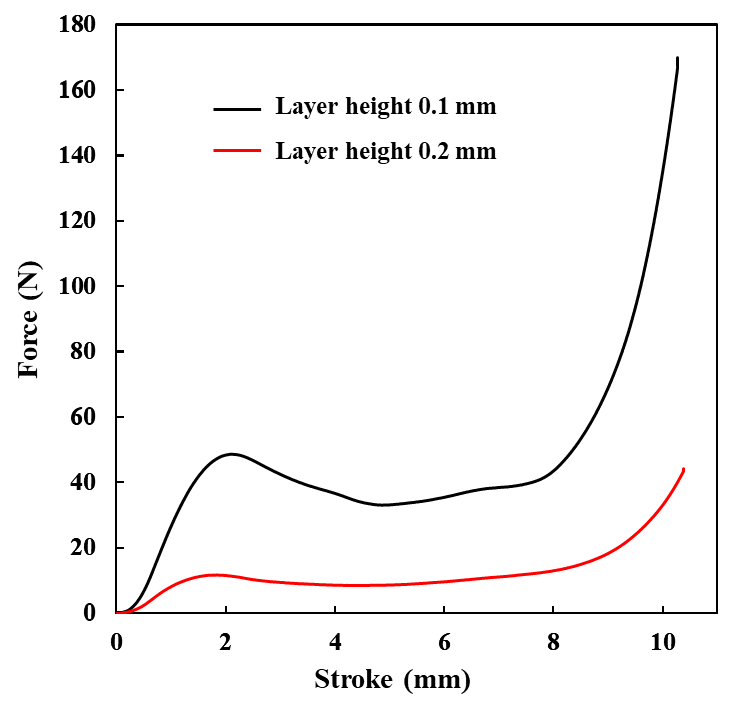
**

**Figure S4. Force vs. Stroke curves of origami blocks prepared by 3D printing with differing layer heights** (0.1 mm in black, 0.2 mm in red)

**
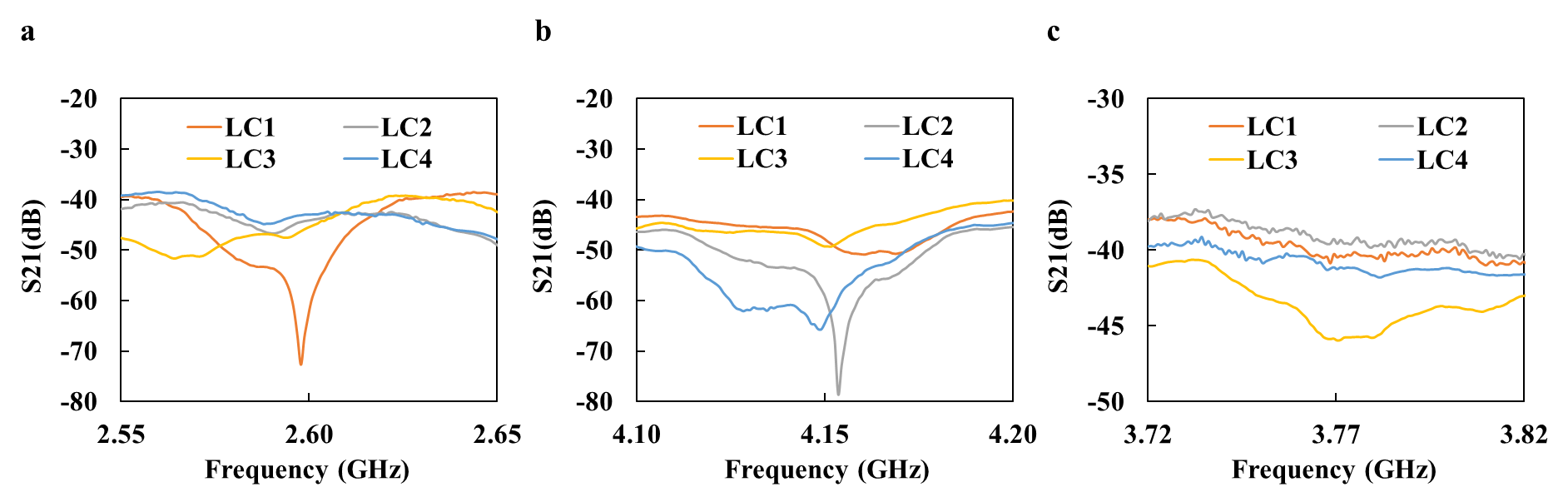
**

**Figure S5 Pressure applied on individual sensors** a) The cylindrical origami block on LC1 was compressed completely b) The cylindrical origami block on LC2 was compressed completely c) The cylindrical origami block on LC3 was compressed completely.

**
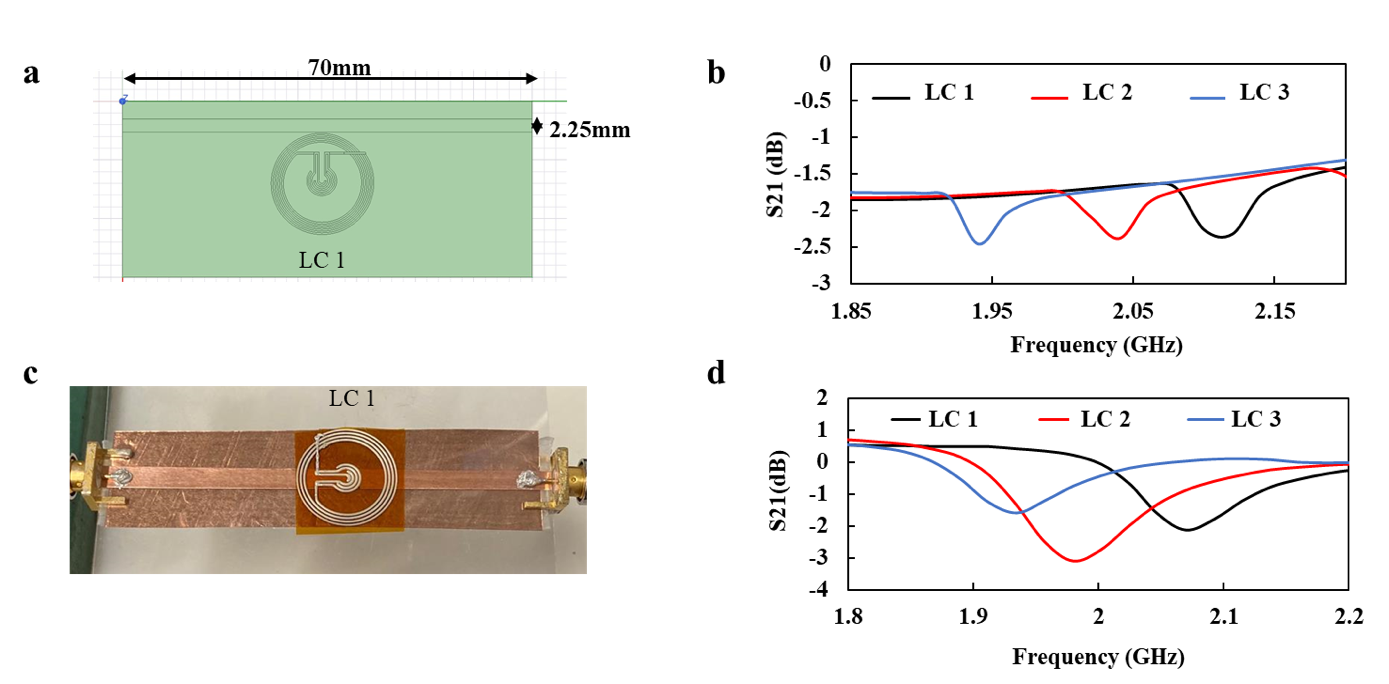
**

**Figure S6. S21 simulation and measurement for sensor 1 with a straight strip line antenna** a) A simulation model of sensor 1 with a straight strip line b) S21 plot of the model of a) c) An experiment set up of sensor 1 with a strip line for the measurement of S21 d) S21 plot of the case of c).

**
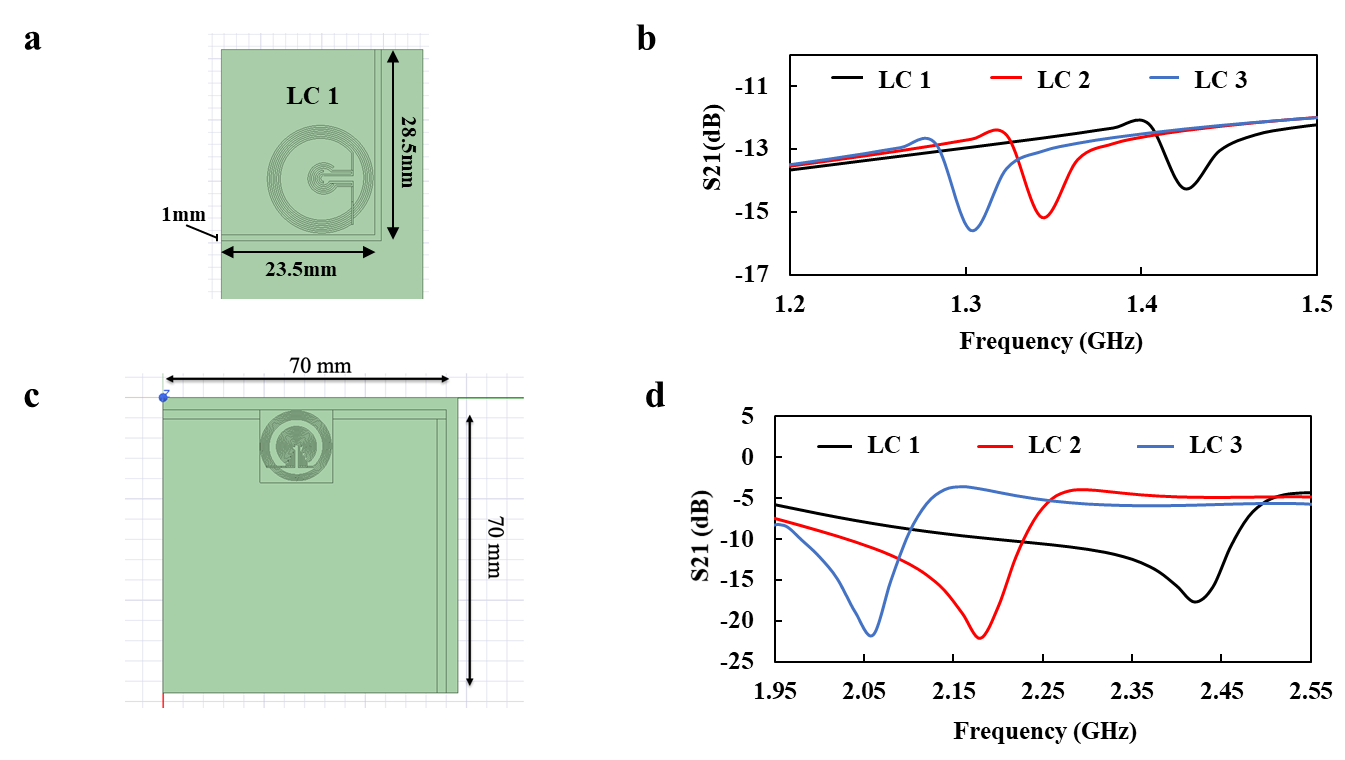
**

**Figure S7. S21 simulation and measurement - L antenna** a) A simulation model of sensor 1 and L antenna b) S21 plot of the simulation result of the model of a) shows distinguishable peaks at 1.30, 1.34, and 1.42 GHz c) A simulation model of sensor 3 and L antenna d) S21 plot of the simulation result of the model of c) shows distinguishable peaks at 2.06, 2.18, and 2.42 GHz e) schematic of experiment set up for S21 measurement of sensor 2 with an L antenna f) S21 plot from the measurement of the case of e).

**
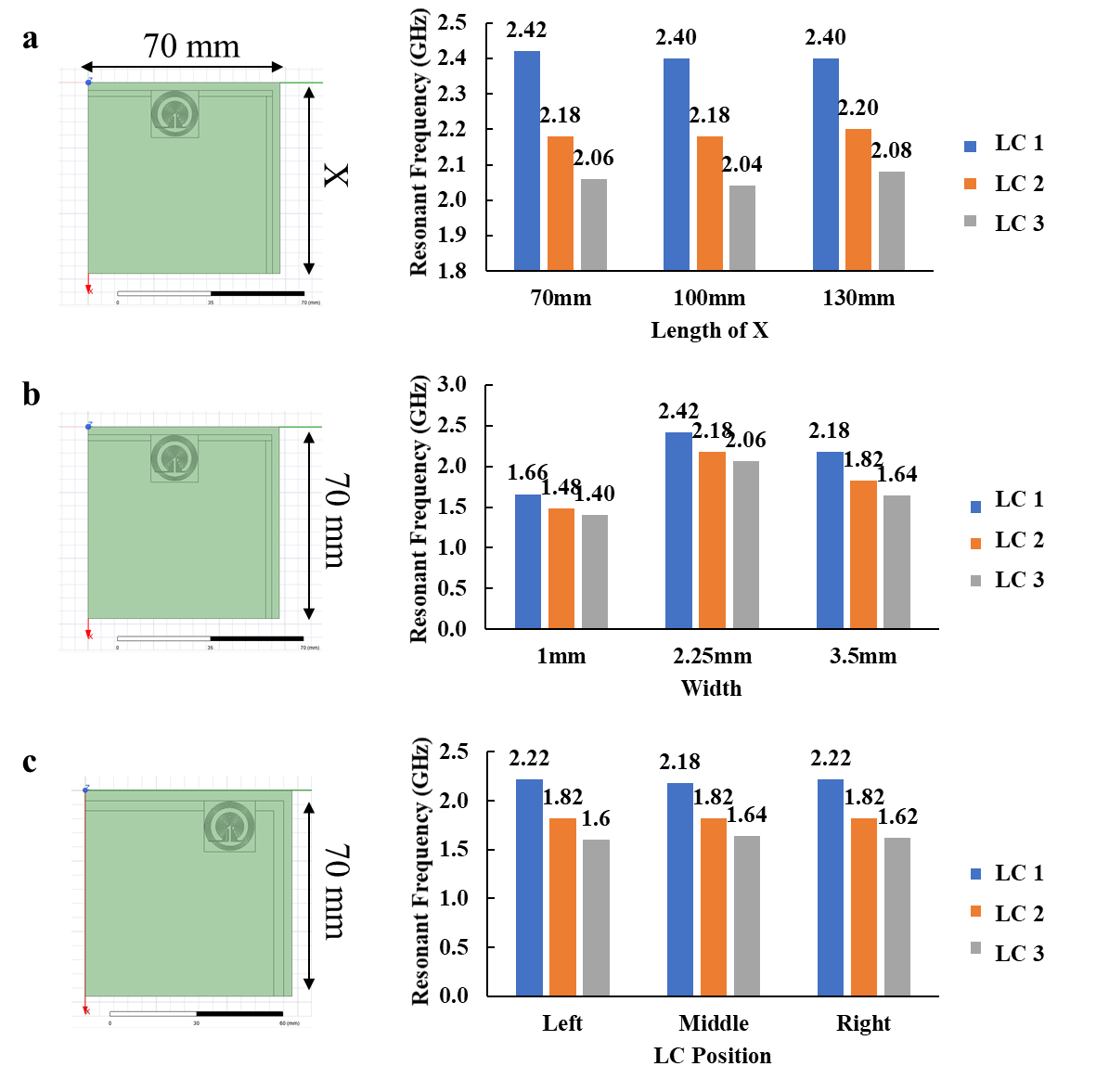
**

**Figure S8. Parametric study of S21 simulation** a) The simulation model and the corresponding result plot of resonant frequency depending on the length of strip line b) The simulation model and the corresponding result plot of resonant frequency depending on width of strip line c) The simulation model and the corresponding result plot of resonant frequency depending on position of LC on top of strip line.
